# Re-evaluating the role of *hok* /Sok in T4 phage defense

**DOI:** 10.17912/micropub.biology.002040

**Published:** 2026-03-20

**Authors:** Adriana Messineo, Andrés Escalera-Maurer, Maria Núria Ramos-Corominas, Anaïs Le Rhun

**Affiliations:** 1 Univ. Bordeaux, CNRS, INSERM, ARNA, UMR 5320, U1212, F-33000 Bordeaux, France

## Abstract

The *hok* /Sok toxin-antitoxin system was shown to protect *Escherichia coli* BK6 from the T4 phage at low multiplicity of infection. One proposed mechanism relies on the rapid depletion of the Sok RNA antitoxin after T4-induced transcriptional arrest, allowing Hok toxin translation. In this model, Hok-mediated cell death prevents phage replication, acting as an altruistic defense system to limit infection spread. Here, we showed that the presence of *hok* /Sok is not sufficient to protect *E. coli* against phage T4 infection. Our findings point to a gap in our understanding of the requirements for phage defense mediated by *hok* /Sok.

**Figure 1. hok /Sok does not provide protection against T4 phage infection:**
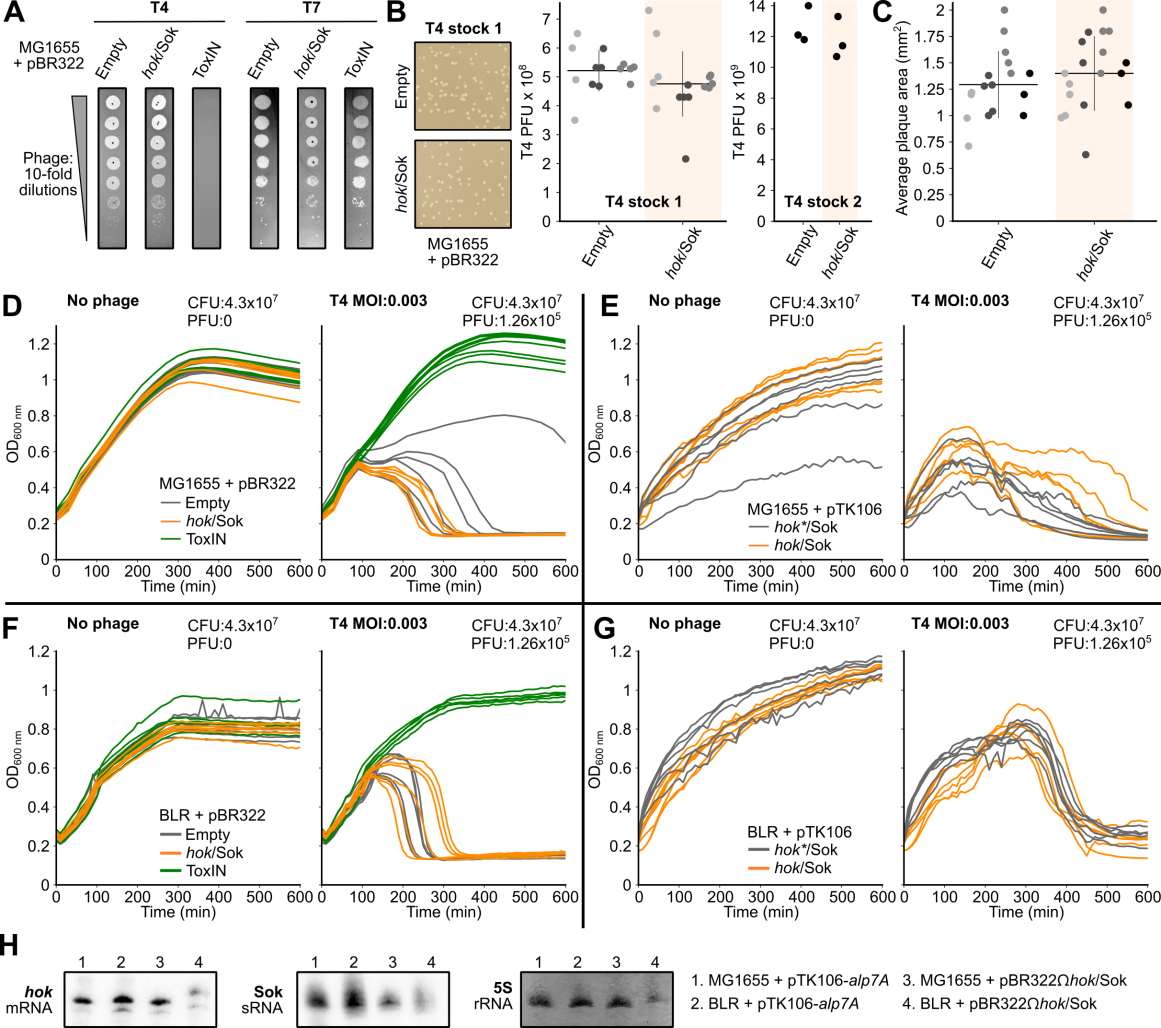
**A.** Drop assay of phage T4 (test) and T7 (control). Serial dilutions of phages T4 and T7 (10 ^-0^ to 10 ^-7^ ) were spotted on a bacterial lawn of *E. coli* MG1655 with the empty pBR322 plasmid or pBR322 encoding either *toxIN* or *hok* /Sok *.* Representative pictures of three independent drop assays are shown. *toxIN,* but not *hok* /Sok, confers protection to *E. coli* MG1655 against phage T4 infection. As expected neither *toxIN* or *hok* /Sok defended against phage T7. **B.** Representative pictures of infected *E. coli* MG1655 lawns in plates carrying either the empty pBR322 or the pBR322Ω *hok* /Sok plasmid (left). Total PFU counts from two T4 phage stocks (middle and right) on bacterial lawns harboring either empty pBR322 or pBR322Ω *hok* /Sok. For T4 stock 1 (middle panel), three biological replicates were done (shown as separate dot columns with different shades of gray), each with five technical replicates (represented by individual dots). Mean and standard deviation are shown. For T4 stock 2 (right panel), one biological replicate with three technical replicates was done. **C.** Area of the plaques from B. A paired-sample t-test was conducted to compare plaque number and size between the two strains, we obtained p-values of 0.5211 and 0.2797 respectively, indicating that the numbers did not differ significantly. **D-G.** Growth curves of *E. coli* MG1655 and BLR strains containing either an empty vector or the corresponding vector encoding *toxIN* , *hok/* Sok or *hok* */Sok (inactive Hok variant). Two different plasmid backbones were used: pBR322 and pTK106 (Table S2). Bacteria were infected at low MOI (0.003). As a control, the culture was grown without phages. Colony forming units (CFU) and phage forming units (PFU) are indicated for each experiment. Colonies from at least three independent plasmid transformations were tested. While protection against T4 phage infection was observed in both strains carrying the plasmid with *toxIN* , we did not observe defense in strains harboring plasmids containing *hok* /Sok. **H.** Northern blot analyses of *hok* and Sok RNAs from total RNA extracted from *E. coli* MG1655 or BLR strains harboring pTKW106- *alp7A or* pBR322Ω *hok* /Sok, both encoding *hok* /Sok. The *hok* mRNA and Sok sRNA were detected using the radioactively labeled probes FB178 and FB213, respectively. 5S rRNA served as a loading control and was stained by methylene blue. *hok* and Sok RNAs were detected in all strains, confirming their expression under our experimental conditions.

## Description

Toxin-antitoxin systems (TAs) are bacterial loci composed of a toxin protein and a cognate antitoxin that functions as an antidote. When these systems are activated, the toxin arrests bacterial growth for the benefit of the population. Three main biological functions of these systems have been described, *ie.* plasmid maintenance (Gerdes et al. 1997; Harms et al. 2018), dormancy in response to a stress (Page and Peti 2016; Ronneau and Helaine 2019) and phage defense (LeRoux and Laub 2022; Lopatina et al. 2020; Song and Wood 2020).

The *hok/* Sok type I toxin-antitoxin system (T1TA) is the only TA from this type reported to mediate phage defense so far (Pecota and Wood 1996). In this system, the Sok antitoxin is a small regulatory RNA that inhibits *hok* toxin mRNA translation by binding to its 5'&nbsp;UTR, leading to RNase&nbsp;III degradation of the RNA duplex. *E. coli* expressing the *hok* /Sok system from the R1 plasmid is partially protected against phage T4, but not phage T7 (Pecota and Wood 1996). Efficiency of plating (EOP) of phage T4 is reduced by 42% in the presence of *hok* /Sok while the plaque size decreases by 85%. In liquid culture, *hok* /Sok-mediated phage defense is observed at a multiplicity of infection (MOI) of 0.001 but not at higher MOI such as 0.1 (Pecota and Wood 1996). The mechanism by which the system is activated upon infection has not been investigated. However, it was proposed that T4-mediated transcriptional shutdown may lead to depletion of the unstable Sok antitoxin, allowing translation of the stable *hok* mRNA. Thus, infected cells expressing *hok* /Sok would be killed before the phage propagates (Pecota and Wood 1996) *.* However, an alternative hypothesis was proposed later, where *hok* /Sok might protect the host by slowing growth and delaying phage development rather than killing the cell (Song and Wood 2018; 2020).

Here we aimed at investigating the mechanism by which the *hok/* Sok system confers protection against phage T4. For that, we first tested the EOP by drop assay ( Fig. 1A ) and plaque assay ( Fig. 1B and C) of phage T4 and T7 on *E. coli* MG1655 strains carrying a plasmid encoding the *hok* /Sok locus from the R1 plasmid, the ToxIN system (a type III TA defending against phage T4 used as a positive control) (Guegler and Laub 2021), or the corresponding empty vector (pBR322). As expected, ToxIN conferred protection against phage T4 but not against phage T7 infection. In contrast, we did not observe any difference in EOP or in the plaque sizes in the strain harboring the *hok* /Sok-encoding plasmid compared to the strain carrying the empty vector ( Fig. 1A, B and C). We also monitored the growth of *E. coli* MG1655 challenged with T4 phages at low MOI (0.003) or without any phages. Two plasmid backbones with different copy numbers (PCN), carrying the *hok* /Sok or ToxIN loci, were used: pBR322 and pTKW106-alp7A (Table 2). We observed ToxIN- but not *hok* /Sok-mediated phage defense upon T4 infection ( Fig. 1D ). Previous studies were carried out in *E. coli* strain BK6 [AMA1004 Δ( *srl* - *recA* )306::Tn10] containing the *hok* /Sok system from the R1 plasmid on pTKW106 (a pUC18-derived plasmid) (Pecota and Wood 1996), which are different to the strains and plasmids that we used. To test whether we could recapitulate the phenotype using other strains, we further evaluated T4 infection on the BLR strain (∆( *srl-recA* )306::Tn10 as the BK6 strain), expressing *hok* /Sok and ToxIN from the pBR322- and pTKW106-derived plasmids. Again, we did not observe phage defense mediated by *hok* /Sok ( Fig. 1E, F and G). As phages rapidly evolve anti-defense mechanisms (Srikant et al. 2022; Subedi and Barr 2021), we tried to rule out differences in the genetic background of the T4 stock used in our study compared to the stock used by Pecota et al. (Pecota and Wood 1996) by testing another T4 stock, originating from the DSMZ collection center ( Fig. 1B and C). Both stocks yield comparable results. Finally, Northern blot analysis confirmed the expression of *hok* and Sok RNAs under our experimental conditions ( Fig. 1H ).

Overall, several parameters could explain the discrepancy between our results and the previous report. 1) differences in experimental conditions, 2) difference in *hok/* Sok expression levels, 3) the genetic background of the host strain or, 4) the genotype of T4 phage. Regarding the experimental conditions, we used very similar bacterial input (4.3x10 ^7^ CFU here compared to 4x10 ^7^ in the original study), a similar MOI (0.003 compared to 0.001 in the original study), the same media (LB), temperature (37°C) and similar agitation (200 vs 250 rpm) (Pecota and Wood 1996). However, in the previous study, the experiment was performed in 20 mL of media in a 250 mL flask while ours was done in 100 µl in a 96-well plate (Pecota and Wood 1996). We used a plasmid derived from the pTKW106 plasmid used in the original study, which differs only by the addition the *alp7* gene that was inserted to ensure equal segregation during bacterial division (Danino et al. 2015). We also used plasmids with a pBR322 backbone, which were previously used in T4 phage defense experiments (Guegler and Laub 2021). Regarding the genetic background of the host, the *E. coli* strain used in the original study was a derivative of MG1655 called AMA1004 while we used MG1655 and BLR strains (Pecota and Wood 1996). The minor differences in conditions or plasmids described above do not explain the observed phenotype, which instead could be attributed to a difference in the host or T4 genotype.

Overall, our findings suggest that the previously attributed phage defense function of *hok* /Sok would be context-dependent. Further studies are needed to elucidate the precise conditions under which *hok* /Sok systems might contribute to phage defense, the exact mechanism of action as well as the reasons behind its inability to protect in our conditions.

## Methods

**Growth conditions**

Bacterial strains (listed in Table 1) were grown in LB at 37°C with an agitation of 200 rpm or LB agar supplemented when needed with 100 μg/mL ampicillin or 50 μg/mL kanamycin unless indicated otherwise.

**Plasmid construction**

To construct LRP24 (pBR322Ω *hok* /Sok), expressing *hok* /Sok, the empty LRP19 (BR322-EV) plasmid was linearized using primers LRO288/289 and digested with DpnI. The *hok* /Sok locus from the R1 plasmid was amplified from LR12 (XTL632 *tetA-sacB* :: *hok* /Sok) genomic DNA (Le&nbsp;Rhun et al. 2022) using primers LRO3/4. Finally, 30 ng of the linearized BR322-EV plasmid and 20 ng of the amplified *hok* /Sok fragment were assembled using 2.5 μl of NEBuilder® HiFi DNA Assembly Master Mix in a final volume of 5 μl, and the reaction was incubated at 50 °C for 1 h. To construct LRP98, containing an inactive *hok* , LRP97 (pTKW106-alp7A, addgene ID 69360; (Danino et al. 2015)) was amplified by site-directed mutagenesis using primers LRO453/454 to introduce the T208G (V12G) mutation in Hok (Le&nbsp;Rhun et al. 2022) and digested by DpnI. Both constructs (pBR322Ω *hok* /Sok and pTKW106alp7AΩ *hok* */Sok) were transformed into *Escherichia coli* Top10 competent cells. Transformants were plated on LB agar supplemented with ampicillin for pBR322Ω *hok* /Sok or kanamycin for pTKW106alp7AΩ *hok* */Sok. For LRP24, colony PCR was performed using primers LRO3/4, and sequencing was carried out with primer LRO4. For LRP98, colony PCR was performed using primers LRO20/24, and sequencing was carried out with primer LRO24. Plasmids and oligonucleotides are listed in Tables 2 and 3.

**Phage drop assay**

A single colony of plasmid-containing *E. coli* MG1655 strains was inoculated in 5 mL of LB supplemented with the appropriate antibiotic and grown overnight. The culture was mixed with an appropriate volume of top agar (10 g/L tryptone, 8 g/L NaCl, 7 g/L agar) to reach a final optical density at 600 nm (OD _600_ ) of 0.6, then poured onto LB agar plates and allowed to dry for 30 minutes. Three µL of ten-fold serial dilutions of phages T4 and T7 (10⁻ ^0^ to 10⁻⁷) were spotted onto the top agar containing the different bacterial strains. After drying at room temperature, plates were incubated for 3 hours for T7 phage infection or overnight for T4 phage infection. Pictures of the plates were taken using the colorimetric OD measurement of the Amersham ImageQuant™ 800 imaging system from Cytiva.

**Phage plaque assay**

An overnight culture of *E. coli* MG1655 strains carrying the desired plasmid was prepared by inoculating a single colony into 5 mL of LB medium supplemented with the appropriate antibiotic. The following day, 100 µL of the overnight culture was mixed with 100 µL of T4 phage dilution between 10⁻ ^7^ and 10⁻⁶ (T4 stock 1) and 10⁻ ^8^ (T4 stock 2) and 2.5 mL of top agar. After drying at room temperature, the plates were incubated overnight. Images were acquired using an Epson perfection V800 photo scanner with the following settings: document source = transparency unit, document type = color positive film, image type = 48-bit color, resolution = 1200 dpi, and scanning quality = high. Plaque number and area were quantified using Fiji and paired-sample t-test was conducted to compare plaque number and size.

**Growth curves**

A single colony harboring the plasmid of interest was inoculated in 5 mL of LB medium supplemented with the appropriate antibiotic and grown overnight. The following day, the culture was diluted 10-fold and grown until it reached an OD₆₀₀ of 0.6. Ninety µL of the diluted culture were mixed with 10&nbsp;µL of diluted phage suspension in a 96-well plate, resulting in a multiplicity of infection (MOI) of 0.003 (1.26x10 ^5^ PFU/mL). Bacterial growth (OD _600_ ) was monitored every 10 min for 16 h using the BioTek Synergy H1 plate reader from Agilent, the BioTek Epoch 2 Microplate Spectrophotometer or the FLUOstar® Omega multi-mode microplate reader, shaking at 600 rpm. Control cultures without phages were added. Data were analyzed and plotted using Jupyter Notebook (Python 3.8, Pandas 2.0.3, matplotlib 3.7.3, seaborn 0.13.2).

**Northern blotting analysis**

10 ml *E. coli* cultures (MG1655 pTKW106- *alp7A* , BLR pTKW106- *alp7A* , MG1655 pBR322Ω *hok* /Sok and BLR pBR322Ω *hok* /Sok,) were harvested at an OD₆₀₀ _nm_ of 0.6 and immediately mixed with 1.25 mL of ice-cold stop solution (95% ethanol, 5% phenol) and pelleted. Cell pellets were resuspended in 500 μL of lysis solution (20 mM sodium acetate pH 5.2; 0.5% SDS; 1 mM EDTA) and mixed with 500 μL of phenol preheated to 65°C. Samples were incubated at 65°C for 10 min and centrifuged at 13 000 rpm for 10 min. The aqueous phase was transferred to a fresh tube containing an equal volume of chloroform/isoamyl alcohol and centrifuged at 13 000 rpm for 10 min. The aqueous phase was collected, and RNAs were precipitated by adding 2.5 volumes of 100% ethanol and 0.1 volume of 3 M sodium acetate (pH 5.2), followed by an incubation at -20°C. RNAs were pelleted by centrifugation at 13 000 rpm at 4°C, washed with 70% ethanol, air-dried, and resuspended in water. RNA concentration was measured using a spectrophotometer, and RNA integrity was assessed by electrophoresis on a 2% agarose gel. Northern blot analysis was performed using 8&nbsp;μg of total RNA separated on 8% polyacrylamide gels containing 7 M urea in 1X Tris-borate-EDTA (TBE) buffer. RNAs were transferred to a nylon membrane (Hybond-N+, Cytiva) by electroblotting in 1X TBE at 8 V overnight at 4°C. RNAs were UV cross-linked to the membrane (302 nm, 2 min). The probes FB178 and FB213 were 5′ end-labelled with ³²P and hybridized to the membrane overday at 42 °C in Church buffer (1 mM EDTA, 0.25 M NaPO₄ pH 7.2, 7% SDS). Membranes were washed twice for 5 min in 2× SSC (Saline–Sodium Citrate) containing 0.1% SDS, and signals were detected after overnight exposure using a Pharos FX phosphorimager (Bio-Rad).

## Reagents

**Table 1. Table of strains**

**Table:** 

**Strain**	**Genotype**	**Available from**
**MG1655**	F⁻ λ⁻ *ilvG⁻ rfb‑50 rph‑1*	Lab collection
**LR12**	XTL632 *tetA-sacB* :: *hok* /Sok	Lab collection
**BLR**	*F– ompT hsdSB(rB– mB– ) gal dcm lac ile (DE3) ∆(srl-recA)306::Tn10 (Tet R)*	Novagen
**Phages**	**Class > Order > Family > Subfamily > Genus**	**Available from**
**T4 stock 1**	*Caudoviricetes > Pantevenvirales > Straboviridae > Tevenvirinae > Tevenvirinae*	Aude Bernheim Lab
**T4 stock 2**		#DSM4505 Leibniz Institute DSMZ- German Collection of Microorganisms and Cell Cultures
**T7**	*Caudoviricetes > Autographivirales > Autotranscriptaviridae > Studiervirinae > Teseptimavirus*	Aude Bernheim Lab

**&nbsp;**

**Table 2. Table of plasmids**

**Table:** 

**Code**	**Plasmid name**	**Genotype**	**Plasmid copy number**	**Source**
LRP19	pBR322-EV	-&nbsp;Empty vector control -&nbsp;Derivative of pBR322 with pTet removed -&nbsp;Ampicillin/Carbenicillin resistant	~ 20	Michael T. Laub lab (Guegler and Laub 2021)
LRP20	pBR322Ω *toxIN*	-&nbsp; *toxIN* under native promoter -&nbsp;Ampicillin/Carbenicillin resistant	~ 20	Michael T. Laub lab (Guegler and Laub 2021)
LRP24	pBR322Ω *hok* /Sok	-&nbsp; *hok* /Sok locus under native promoter -&nbsp;Ampicillin/Carbenicillin resistant	~ 20	This study
LRP97	pTKW106- *alp7A*	- *hok* /Sok locus under native promoter -&nbsp;Kanamycin resistant	~ 80 - 100	Addgene ID69360 (Danino et al. 2015)
LRP98	pTKW106alp7AΩ hok* /Sok	-&nbsp;Inactive *hok* carrying T208G (V12G) mutation (Le&nbsp;Rhun et al. 2022) -&nbsp;Kanamycin resistant	~ 80 - 100	This study

&nbsp;

**Table 3. Table of oligonucleotides**

**Table:** 

**Code**	**Name**	**Sequence**	**Description**
LRO288	pBR322-EV_F	atgcgtaagcattgctgttgaagaattggagccaatcaattc	Primers to amplify the pBR322-EV plasmid and introduce flanking regions for *hok* /Sok locus insertion
LRO289	pBR322-EV_R	tcggtgtttgctggtgatttacatgagaattcttgaagacg	
LRO3	*hok* /Sok_F	aaatcaccagcaaacaccga	Primers used to amplify the *hok/* Sok locus from the LR12 gDNA to be inserted in pBR322-EV, for colony PCR and sanger sequencing (LRO4 only)
LRO4	*hok* /Sok_R	caacagcaatgcttacgcata	
LRO453	pTKW106- *alp7A* _ T208G_F	gtctggtgtg **G** gttgatcgt	Primers used to perform PCR to insert the *hok* *T208G (V12G) mutation that inactivates the Hok toxin in the pTKW106- *alp7A* plasmid. The mutation is indicated in bold uppercase
LRO454	pTKW106- *alp7A* _ T208G_R	acgatcaac **C** cacaccagac	
LRO20	pTKW106 *alp7A* _F	gcagaaagaagatagccccg	Primers used for colony PCR and Sanger sequencing (LRO24 only) of the pTKW106 *alp7A* Ω *hok* */Sok plasmid after insertion of the T208G_ V12G mutation
LRO24	pTKW106 *alp7A* _R	gcttcagtagtcagaccagcat	
FB178	*hok_* NB	cggcaacaaaccaccttcac	Oligoprobe used to target the *hok* mRNA by Northern blot
FB213	Sok_NB	aggcatccctatgtctagtc	Oligoprobe used to target the Sok sRNA by Northern blot
